# Supplementary material for: A 29-mRNA host response test from blood accurately distinguishes bacterial and viral infections among emergency department patients
Source: Intensive Care Med Exp. 2021 Jun 18;9:31. doi: 10.1186/s40635-021-00394-8 (PMC8211458; doi:10.1186/s40635-021-00394-8)
Supplement: Supplementary file 1 — Additional file 1. Additional figures and tables. [file 40635_2021_394_MOESM1_ESM.docx]

**A 29-mRNA Host Response Test from Blood Accurately Distinguishes Bacterial and Viral Infections Among Emergency Department Patients**

Asimina Safarika^1^, James W. Wacker^2^, Konstantinos Katsaros^3^, Nicky Solomonidi^1^,

George Giannikopoulos^4^, Antigone Kostaki^1^, Ioannis M. Koutelidakis^5^, Sabrina M. Coyle^2^, Henry K. Cheng^2^, Oliver Liesenfeld^2^, Timothy E. Sweeney^2^, Evangelos J. Giamarellos-Bourboulis^1^

Affiliations:

^1^4th Department of Internal Medicine, National and Kapodistrian University of Athens, Greece;
^2^Inflammatix Inc, Clinical Affairs, Burlingame, CA, United States;

^3^Department of Surgery, Nafplion General Hospital, Greece;

^4^Department of Internal Medicine, Syros General Hospital, Greece;

^5^2^nd^ Department of Surgery, Aristotle University of Thessaloniki, Greece

* To whom correspondence should be addressed:

Evangelos J. Giamarellos-Bourboulis, MD, PhD, FISAC

Professor of Internal Medicine

4^th^ Department of Internal Medicine

ATTIKON University Hospital

1 Rimini Str

12462 Athens

Greece

**Supplementary Table S1** Linear Regression with InSep Bacterial Score as Dependent Variable.

Linear regression revealed that other than lactate the markers listed are not influencing the InSep score.

| Coefficients: | Estimate | Std. Error | t | P |  |
| --- | --- | --- | --- | --- | --- |
| (Intercept) | -0.007 | 0.102 | -0.069 | 0.945 |  |
| Consensus Adjudication: bacterial | 0.435 | 0.050 | 8.768 | 0.000 | *** |
| Age | 0.001 | 0.002 | 0.776 | 0.442 |  |
| Sex - Male | 0.021 | 0.045 | 0.459 | 0.648 |  |
| Immunocompromised - Yes | -0.066 | 0.047 | -1.391 | 0.171 |  |
| Lactate | 0.062 | 0.024 | 2.614 | 0.012 | * |

**Supplementary Table S2** Linear Regression with InSep Viral Score as Dependent Variable. Linear regression revealed that other than lactate the markers listed are not influencing the InSep score.

| Coefficients: | Estimate | Std. Error | t | P |  |
| --- | --- | --- | --- | --- | --- |
| (Intercept) | 0.227 | 0.154 | 1.473 | 0.148 |  |
| Consensus Adjudication: viral | 0.438 | 0.070 | 6.257 | 0.000 | *** |
| Age | 0.001 | 0.002 | 0.291 | 0.772 |  |
| Sex - Male | -0.022 | 0.062 | -0.355 | 0.724 |  |
| Immunocompromised - Yes | -0.012 | 0.065 | -0.188 | 0.852 |  |
| Lactate | -0.071 | 0.033 | -2.188 | 0.034 | * |

**Supplementary Figures**

**A**

**
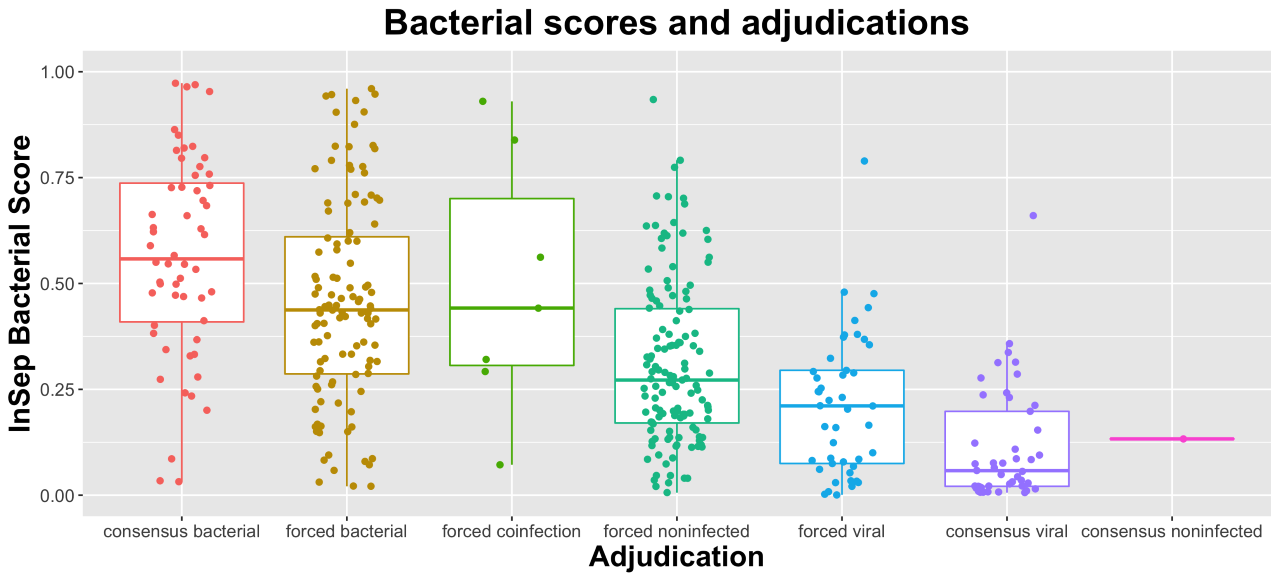
**

**B**


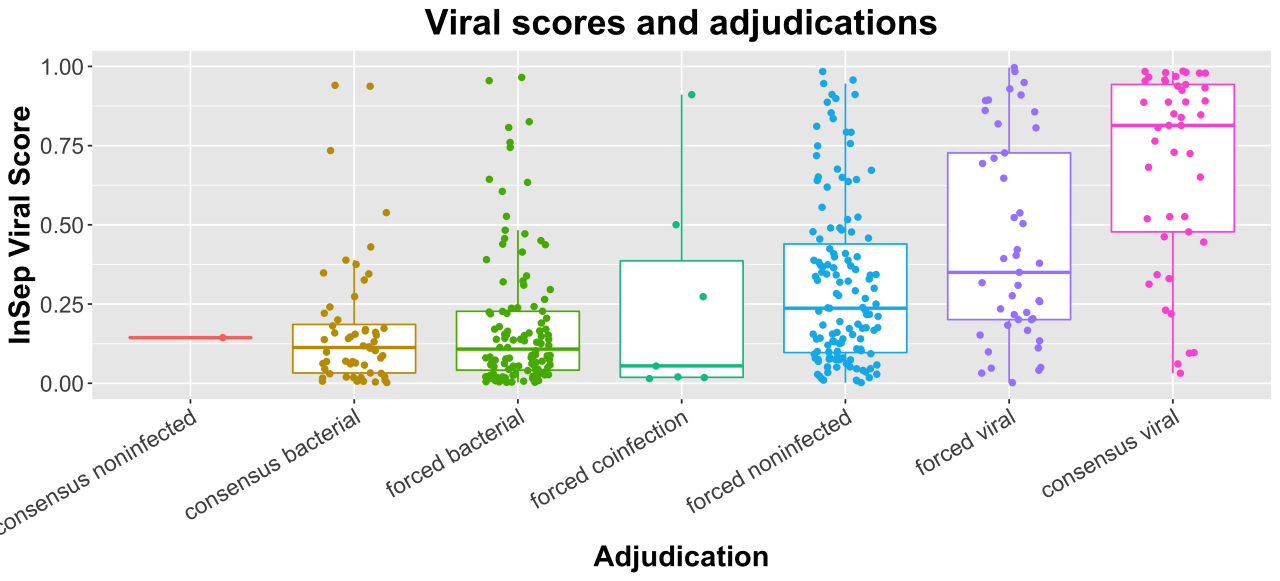


**Supplementary Figure S1:** BVN-2-generated InSep bacterial (A) and viral (B) scores grouped by adjudication status.

**Supplementary Fig. S2** InSep concordance with results of bacterial and viral pathogen detection tests. InSep results segmented by interpretation bands compared to conventional diagnostic test results for the presence of (A) bacterial and (B) viral pathogens.
Interpretation of positive blood cultures to determine clinically significant pathogens was based on guidance published by clinical microbiology and infectious disease specialists (18, 20). Urine cultures that grew pathogenic bacteria with a colony count of >10^5^/ml were considered significant (19). In respiratory specimens, the following viruses were considered clinically significant: influenza A and B, parainfluenza 1-4, RSV, human metapneumovirus and SARS.

**Supplementary Fig. S3** InSep performance in immunocompromised patients. InSep (A) bacterial scores and (B) viral score results segmented by interpretation bands and immune status in the 20 (out of 102) consensus adjudicated patients who are immunocompromised. (C) shows the same boxplots as in Figure 2) but with immunocompromised patients colored in green and dotted red lines which separate the four interpretation bands.
